# Endourological *versus* open cystolithotomy for bladder stone management among children: A systematic review and meta-analysis

**DOI:** 10.12688/f1000research.129270.1

**Published:** 2023-02-02

**Authors:** Randy Fauzan, Hendra Herman, Wendy Rachman, Ardiansyah Periadi Sitompul, Ari Astram

**Affiliations:** 1 Urology Division, Department of Surgery, School of Medicine, Raden Mattaher Hospital, Universitas Jambi, Jambi, Jambi, 36121, Indonesia; 2 Urology Division, Department of Surgery, School of Medicine, Universitas Jambi, Abdul Manap Hospital, Jambi, Jambi, 36129, Indonesia; 3 Urology Division, Department of Surgery, School of Medicine, Kandou Hospital, Universitas Sam Ratulangi, Manado, Manado, 95163, Indonesia

**Keywords:** Endourological techniques, Open surgery, bladder stone, children

## Abstract

**Background:** The treatment of choice for bladder stones in children remains debatable. This study aimed to compare the outcomes of endourological and open cystolithotomy for the management of bladder stones in children.

**Methods:** The Medline, Embase, Cochrane controlled trial databases and clinicaltrials.gov were searched for relevant English-language publications from 1 to 30 August 2022. Stone-free rate (SFR), complication rate, length of stay, and procedure duration were compared. Children (male and female) <18 years of age of any ethnicity with bladder stones (single/multiple) were included. Patients with a history of bladder augmentation or diversion were excluded. The quality of studies included was assessed using Cochrane’s Risk of Bias Assessment. The meta-analysis was performed using RevMan.

**Results:** Five articles (436 participants) that compared endourological *versus* open cystolithotomy were included in qualitative and quantitative analyses. Four were non-randomised, retrospective, and single centre studies. While the other one was a randomised controlled trial. Measure outcome characteristics included SFR, complications, procedure duration, and length of hospital stay. There was no significant difference in the SFR between transurethral cystolithotripsy (TUCL) and percutaneous cystolithotomy (PCCL) ( *p=*0.22). There were also no significant differences in complications (TUCL *versus* PCCL, *p=*0.18; TUCL *versus* open cystolithotomy [CL] and PCCL *versus* CL, *p=*0.08). PCCL featured a longer procedure duration than TUCL ( *p<*0.00001), while CL was shorter than TUCL and PCCL (both *p<*0.00001). Finally, in terms of length of stay, TUCL was superior to PCCL and CL, while PCCL was better than CL (all *p<*0.00001).

**Conclusions:** Endourological and open surgical management of bladder stones in children showed comparable SFR and fewer complications. Open surgery offers a shorter procedure duration than endourological management, but PCCL features a shorter procedure duration than TUCL. In terms of length of stay, TUCL and PCCL were superior to CL, while TUCL was better than PCCL.

## Introduction

Bladder stones account for 5% of urolithiasis cases, which are traditionally classified as primary idiopathic, secondary, or migrant. ^1^ In some cases, it can occur in children, especially in developing countries, due to the high prevalence of urinary tract infections and poor nutritional status, especially owing to a protein-poor diet. The incidence in male children is 10-fold that in female. ^2^^,^^3^ The best option for managing these patients was traditionally open cystolithotomy (CL) owing to its high stone-free rate (SFR); however, it results in significant complications, such as long scars, prolonged catheterisation, extended hospitalisation, and risk of infection. ^4^^,^^5^

Due to technological advancements and the application of laser science in medicine, endourological procedures (transurethral cystolithotripsy [TUCL] and percutaneous cystolithotomy [PCCL]) have become popular among urologists. ^6^^,^^7^ Large and hard stones can be disintegrated and removed in large fragments to enable a quick intervention. It is also more advantageous than open surgery in terms of cosmetic outcomes and length of hospital stay. Some studies have shown its safety and efficacy; however, more data are still needed. ^8^^,^^9^

To date, only a few studies have compared endourological procedures and open cystolithotomy for the management of bladder stones in children; therefore, we performed this systematic review and meta-analysis. There were no expected sex and/or gender differences in this study.

## Methods

### Eligibility criteria

The inclusion criteria in this study were as follows: 1) All randomised controlled trials (RCTs) and comparative non-randomised studies (NRSs) comparing open cystolithotomy and endourological procedures for bladder stones; 2) full-text articles published in English; 3) children (male and female participants) <18 years of age of any ethnicity with bladder stones (single/multiple) were included. Patients with a history of bladder augmentation or diversion were excluded. All studies were grouped into three groups: group A (TUCL *versus* PCCL), group B (TUCL *versus* CL) and group C (PCCL *versus* CL).

### Information sources

We conducted the systematic review in accordance with the Preferred Reporting Items for Systematic Reviews and Meta-analyses (PRISMA) checklist ^26^ and Cochrane Handbook for Systematic Reviews of Interventions. This study followed the SAGER guidelines for reporting sex and gender. ^27^ The MEDLINE (RRID:SCR_002185), EMBASE (RRID:SCR_001650), and Cochrane Central Register of Controlled Trials (RRID:SCR_006576) databases and ClinicalTrials.gov (RRID:SCR_002309) were searched for relevant English-language publications from 1 to 30 August 2022.

### Search strategy

We used keywords adjusted to search engine specification in the form of (bladder stone OR bladder calculi) AND (transurethral cystolithotomy OR percutaneous cystolithotripsy OR open cystolithotomy) AND (children OR paediatric).

### Selection process

The PICOS framework was used to trace studies and identify the suitability of any we found. ^10^ Study selection was carried out independently and duplicated by each author, referring to inclusion and exclusion criteria. The decision to study eligibility was determined by each author independently. Any disagreement was resolved by discussion.

### Data collection process

Data collection was carried out by each author independently and in duplication. We extracted the study’s primary characteristics, including the first author, location, sample size, and publication year. We also extracted patient baseline data and postoperative data, including a SFR, complication rate, procedure duration and length of stay. We used a 2x2 contingency table to obtain each study’s odds ratio (OR) and pooled the overall ORs using RevMan (RRID:SCR_003581) version 5.3.

### Data items

The following data were extracted from each study included in the review: study methodology (study design, year); participant characteristics (age, sex, stone size, lithotripsy energy, SFR, complication, procedure duration, catheterisation, hospital stay and follow up); intervention and comparator description (TUCL, PCCL and CL); and outcome measures.

### Study risk of bias assessment

Risk of bias assessment was independently assessed by each author using the risk of bias assessment tool by Higgins *et al.*, (2011) ^11^ including: random sequence generation, allocation concealment, blinding of participants and personnel, blinding of outcome assessment, incomplete outcome data, selective reporting and other bias, which were produced *via* RevMan (RRID:SCR_003581) version 5.3.

### Effect measures

The odds ratio (OR) with 95% CI was used to assess the success of treatment (SFR and complication). The mean duration and mean hospital stay were assessed using mean difference (MD) with 95% confidence intervals (CI).

### Synthesis methods

The meta-analysis was performed using RevMan (RRID:SCR_003581) version 5.3. The results are described as odds ratios (OR) with 95% confidence intervals (CI) for dichotomous variables and as mean difference with 95% CI for continuous variables. A chi-squared statistic (Cochrane Q) was used to evaluate the level of heterogeneity. The I ^2^ statistic was used to determine the percentage of the total variation in the estimated effect across studies. The data was analysed using the random effect model when I ^2^ >25%, and fixed effect model when I ^2^ is less than 25%. Statistical significance was set at values of *p*<0.05. For studies that provided the minimum and maximum value instead of standard deviation (SD) for the mean difference analysis, estimated SD were calculated with the formula derived from a study by Walter and Yao (2007). ^12^ Sensitivity analyses were not conducted to explore the effect of study quality as there were too few studies and some studies used different criteria for measuring outcomes.

### Reporting bias assessment

We could not use funnel plots of between-treatment effect and its precision on individual studies for publication bias due to the small numbers of studies.

### Certainty assessment

To assess certainty (or confidence) in the body of evidence for an outcome, this meta-analysis used GRADE score, which has four level of evidence very low, low, moderate, and high. ^13^

## Results

### Study selection

**Figure 1.**
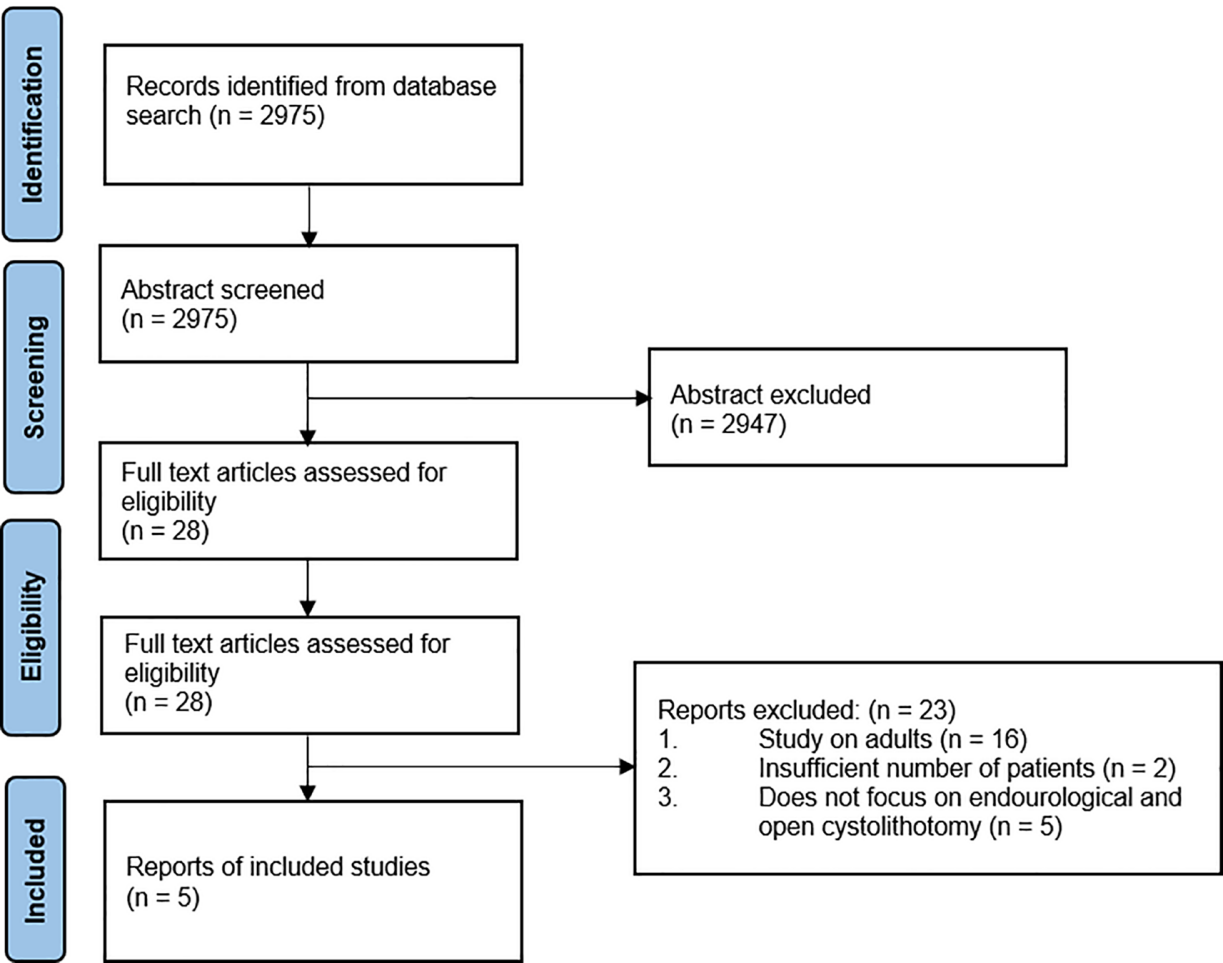
PRISMA flowchart detailing the article identification, screening, and inclusion process and results.

A total of 2,975 articles were initially retrieved from the database. After removing duplicates and evaluating the title and abstracts, 28 articles were subjected to full-text review. From the 28 article studies screened, 23 studies were excluded due to the following reasons: they were studies on adults (n=16), had an insufficient number of patients (n=2), or did not focus on endourological and open cystolithotomy (n=5). Finally, five studies were included in the analysis: four NRS and one RCT. The article selection process was performed according to the PRISMA statement ( Figure 1).

### Study characteristics

Three of the five studies included in this systematic review were non-randomised studies, retrospective, and single centre. ^14^^–^^16^ The other two were prospective, but one was randomised and the other one was not randomised. ^17^^–^^18^ The numbers of patients enrolled in the studies did not differ between studies. The total number of patients in this meta-analysis was 436 patients. Two studies compared endourological (TUCL; PCCL) *versus* open cystolithotomy, two studies compared PCCL *versus* TUCL, and the last one compared PCCL *versus* open cystolithotomy. Measure characteristics included intervention type, sex, age, stone size, and lithotripsy energy. Outcomes (SFR, complications, procedure duration, and length of hospital stay) and follow-up were also measured ( Table 1).

**Table 1.** Paediatric study characteristics. NRS, non-randomised study; RCT, randomised controlled trials; TUCL, transurethral cystolithotripsy; PCCL, percutaneous cystolithotomy; CL, cystolithotomy; yo, years old; mo, months; NR, not reported; XR, X-ray; USG, ultrasonography; w, weeks; Pneu, pneumatic.

Article	Study design/total sample	Intervention	Patient characteristics			Stone size	Lithotripsy energy	Outcome					Follow-up	
			Age (mean ± SD)	Male	Female			Stone- free rate (%)	Complications rate, n (%)	Procedure duration (min)	Catheterisation (days)	Hospital stay (days)	Modality	Duration
Javanmard (2018), Iran ^15^	NRS (N= 146)	TUCL (n=27)	9 ± 5 (1.5–1.7) yo	22	NR	2.54 ± 0.8 (1–4.5) cm	Holmium-YAG laser	100	5 (18.5) late	36.30 ± 5.97 (28–54)	1	1.3 ± 0.66 (1–4)	USG	2 w
		PCCL (n=39)	9.2 ± 5 (1.5–17) yo	34	NR	2.6 ± 1 (1–4.9) cm			5 (12.8) late	30.54 ± 5.27 (22–42)	1 (cystostomy)2 (urethral catheter)	2.49 ± 0.72 (2–4)		
		CL (n=80)	77.6 ± 5.1 (0.5–17.5) yo	73	NR	2.89 ± 1.17 (1–6.2) cm			10 (12.6) late	26.06 ± 6.32 (15–48)	5	3.55 ± 1 (2–8)		
Al-Marhoon (2009), Egypt ^14^	NRS (N=107)	TUCL (n=27)	6.5 ± 4.1 yo	96	11	1.2 ± 0.6 cm	Swiss LithoClast, ^17^ Laser ^10^	100	1 (3.7) operative 1 (3.7) late	46 ± 14	NR	4.8	USG, XR	12 mo
		PCCL (n=27)	7.2 ± 3.8 yo			1.8 ± 0.8 cm	Swiss LithoClast, ^20^ Ultrasound ^7^		1 (3.7) 3 (11) (early)		1 (cystostomy) 2 (urethral catheter)			
		CL (n=53)	5.9 ± 4.6 yo			3.1 ± 1.6 cm	-		1 (1.9) operative	38 ± 12	NR	2.6		
Mahran (2000), Egypt ^16^	NRS (N=52)	TUCL (n=11)	6.3 ± 4.1 yo	11	0	1.2 ± 0.7 cm	Swiss LithoClast, ^7^ Laser ^4^	100	1 (10) operative 1 (10) late	46 ± 14	NR	1.6 ± 0.8	NR	NR
		PCCL (n=16)	3.8 ± 1.6 yo	16	0		Swiss LithoClast, ^12^ ultrasound ^4^		3 (19) early					
		CL (n=25)	0.9 ± 0.6 yo	25	0		-		1 (4) operative	38 ± 12		33.9 ± 1.2		
Shahat (2022), Egypt ^17^	RCT (N= 100)	TUCL (n=50)	36 (4–120) mo	50	0	11 (5–25) mm	Pneumatic (40(85.1)) Holmium laser 7 (14.9)	96	11 (22)	34.2 ± 24.8	2 (Urethral catheter)	3.3 ± 1.4	USG	1 week
		PCCL (n=50)	36 (12–144) mo	50	0	10 (6–26) mm	Pneumatic 19(86.4) Holmium laser 3 (13.6)	98	5 (10)	25.2 ± 18.7				
Mishra (2020), India ^18^	NRS (n=31)	TUCL (n=15)	3.8 ± 0.7 (3–5) yo	15	0	1.3 ± 0.5 (0.8–2.9) cm	Ho-YAG laser 20-40 watts	86.6	2 (13)	38.2 ± 6.7 (35–62)	1 (urethral catheter)	2	USG, uroflowmetry	1 mo, 6 mo
		PCCL (n=16)	2.4 ± 0.9 (1–5) yo	16	0	1.9 ± 0.9 (1–3.6) cm		100	0 (0)	33.5 ± 8.4 (21–51)				
		TUCL pneu (n=13)	7 ± 2.6 yo	13	0	1.7 ± 0.6 cm	Pneumatic		7 (13.5)	36.6 ± 8.7		1.8 ± 0.8		

### Risk of bias in studies

**Figure 2.**
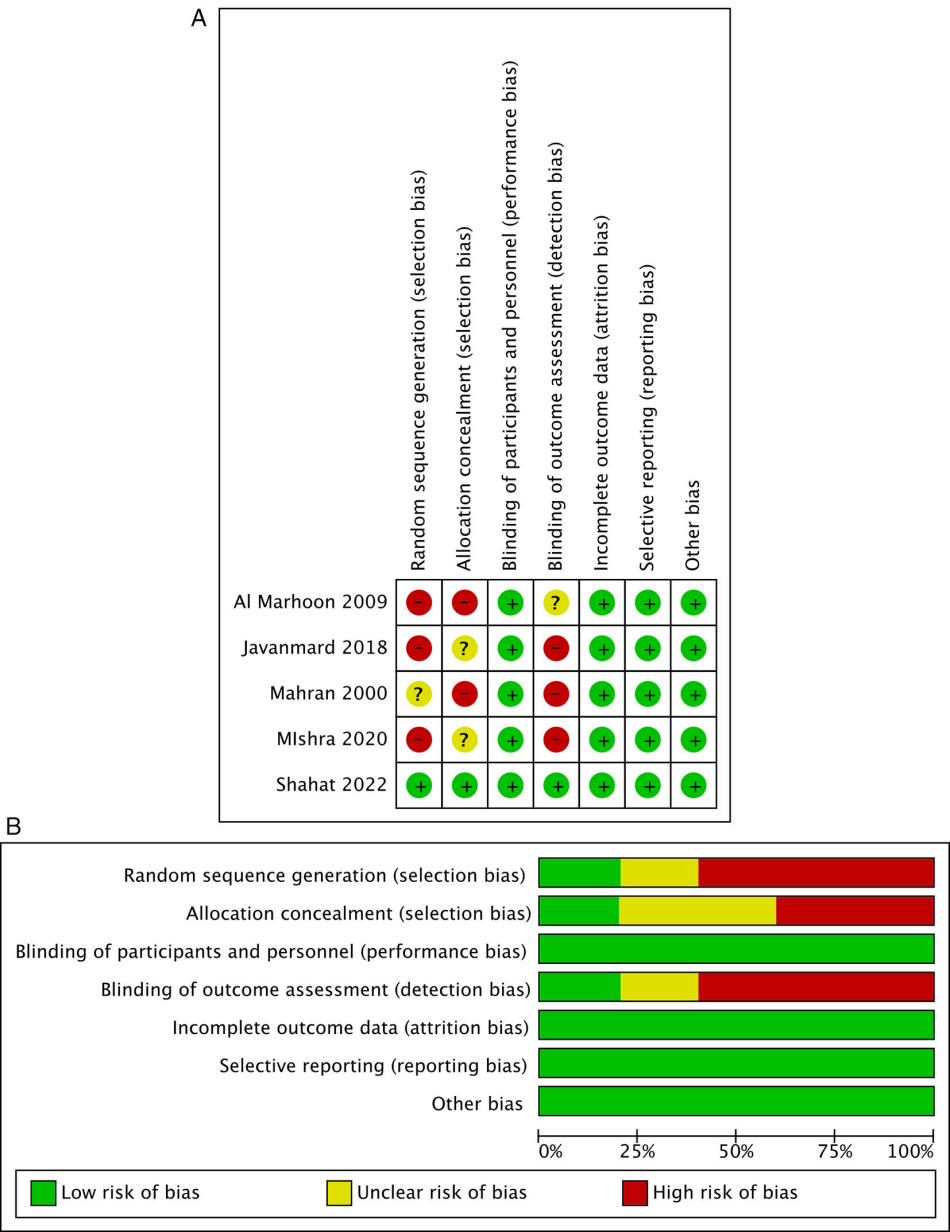
Risk of bias assessment summary using traffic light and summary plots. (A) Traffic light plot and (B) summary plot.

Of the five studies included in this meta-analysis, three had high risk of bias because assessor outcome blinding was not performed. ^14^^,^^15^^,^^18^ Selection bias in some studies was also considered high because no allocation concealment was performed. In general, the quality of the studies in this meta-analysis varied from low to high. The bias assessment was carried out independently by each author and in duplication, in which all authors were resolved by discussion or consensus. Figure 2 visualizes the summary of bias risk.

### Results of individual studies

*Stone-free rate*

Group A (TUCL *versus* PCCL)

Mahran *et al.*, 2000, Al-Marhoon *et al.*, 2009 and Javanmard *et al.*, 2018 reported SFRs of 100%. All stones were removed successfully. ^14^^–^^16^ Mishra *et al.*, 2020 reported both groups (TUCL and PCCL) were comparable with good outcomes. The clearance rate was 100% in the PCCL group, while in the TUCL group it was 86.6%. However statistical significance was not reached. ^18^ Shahat *et al.*, 2022 reported TUCL SFR was 96%, while PCCL 98%. There were no significant differences between both groups ( *p=*1). ^17^

Group B (TUCL *versus* CL) and Group C (PCCL *versus* CL)

Mahran *et al.*, 2000, Al-Marhoon *et al.*, 2009 and Javanmard *et al.*, 2018 reported SFRs of 100%. ^14^^–^^16^

*Complication*

Group A (TUCL *versus* PCCL)

Mahran *et al.*, 2000 reported one (4%) patient who was observed for urethral rupture and extravasation during TUCL and four (15%) patients who developed early and late complications (persistent leakage of urine, acute abdomen, and stricture urethra) for PCCL. ^16^ Shahat *et al.*, 2022 reported that complication for TUCL were persistent haematuria, urethral abrasion, difficulty after catheter removal, and bladder perforation, while PCCL had fever, persistent haematuria, stone entrapped, subcutaneous fluid collection, and intraperitoneal collection. ^17^ Javanmard *et al.*, 2018 reported complications in PCCL included surgical site infection, ^2^ urinary leakage, ^1^ and irritative symptoms, ^2^ while six patients from TUCL group developed haematuria. ^15^ Mishra *et al.*, 2000 reported zero complications in the PCCL group and one patient conversion into open surgery in the TUCL group. ^18^ Al-Marhoon *et al.*, 2009 showed urethral rupture (1 (1.4%)) in the TUCL group and bladder perforation (1 (1.4%)) in the PCCL group. ^14^

Group B (TUCL *versus* CL)

Mahran *et al.*, 2000 reported one (4%) patient who was observed for urethral rupture and extravasation during TUCL and one (4%) patient who had small intestinal injury in the CL group. ^16^ Javanmard *et al.*, 2018 reported six patients from the TUCL group who developed haematuria and one patient who underwent CL had urinary leakage and perivesical fluid collection. ^15^ Al-Marhoon *et al.*, 2009 showed one (1.4%) patient in the TUCL group who had a urethral rupture and one (1.9%) patient who had peritoneal perforation and small intestinal injury during tube drain fixation in the CL group. ^14^

Group C (PCCL *versus* CL)

Mahran *et al.*, 2000 reported four (15%) patients who developed early and late complications (persistent leakage of urine, acute abdomen, and stricture urethra) for PCCL and one (4%) patient who had small intestinal injury in the CL group. ^16^ Javanmard *et al.*, 2018 reported complications in PCCL included surgical site infection, ^2^ urinary leakage, ^1^ and irritative symptoms ^2^ and one patient who underwent CL had urinary leakage and perivesical fluid collection. ^15^ Al-Marhoon *et al.*, 2009 reported one (1.4%) patient had bladder perforation in the PCCL group and one (1.9%) patient had peritoneal perforation and small intestinal injury during tube drain fixation in the CL group. ^14^

*Procedure duration*

Group A (TUCL *versus* PCCL)

Mahran *et al.*, 2000 reported no significant differences in procedure duration between two groups. It was 46 ± 14 minutes for the TUCL or PCCL group. ^16^ Shahat *et al.*, 2022 reported PCCL had significantly faster procedure duration than TUCL ( *p*=0.012). It was 34.2 ± 24.8 minutes for TUCL group and 25.3 ±18.7 minutes for PCCL group. ^17^ Javanmard *et al.*, 2018 reported significantly longer procedure duration in the TUCL group (36.3 ± 5.9 minutes) than the PCCL group (30.5 ± 5.2 minutes) ( *p=*0.000). ^15^ Mishra *et al.*, 2000 reported faster procedure duration for the PCCL group (33.5 ± 8.4 minutes) than the TUCL group (38.2 ± 6.7 minutes). ^18^ Al-Marhoon *et al.*, 2009 reported that the procedure duration was comparable in both groups (46 ± 14 minutes). ^14^

Group B (TUCL *versus* CL)

Mahran *et al.*, 2000 reported no significant differences in procedure duration between the two groups. It was 46 ± 14 minutes and 38 ± 12 minutes in the TUCL and CL groups, respectively. ^16^ Javanmard *et al.*, 2018 reported that the CL group had significantly faster procedure duration (26.0 ± 6.3 minutes) than the TUCL group (36.3 ± 5.9 minutes) ( *p=*0.000). ^15^ Al-Marhoon *et al.*, 2009 reported that procedure duration was comparable in both groups, 46 ± 14 and 38 ± 12 minutes for TUCL and CL, respectively. ^14^

Group C (PCCL *versus* CL)

Mahran *et al.*, 2000 reported comparable procedure duration in both groups. It was 46 ± 14 minutes in the PCCL group and 38 ± 12 minutes in the CL group. ^16^ Javanmard *et al.*, 2018 reported that the CL group (26.0 ± 6.3 minutes) had significantly faster procedure duration than the PCCL group (30.5 ± 5.2 minutes) ( *p=*0.000). ^15^ Al-Marhoon *et al.*, 2009 reported that procedure duration was comparable in both groups, 46 ± 14 and 38 ± 12 minutes in the PCCL and CL groups, respectively. ^14^

*Length of hospital stay*

Group A (TUCL *versus* PCCL)

Mahran *et al.*, 2000 reported that length of stay was comparable in both groups (1.6 ± 0.8 days). ^16^ Shahat *et al.*, 2022 reported no significant difference in terms of length of stay for both groups, 3.2 ± 1.4 and 3.2 ± 1.4 days ( *p=*0.13) in the TUCL and PCCL groups, respectively. ^17^ Javanmard *et al.*, 2018 reported that the TUCL group (1.3 ± 0.6 days) had shorter length of stay than the PCCL group (2.4 ± 0.7 days) ( *p=*0.001). ^15^

Group B (TUCL *versus* CL)

Javanmard *et al.*, 2018 reported that the TUCL group (1.3 ± 0.6 days) had shorter length of stay than the CL group (3.5 ± 1 days) ( *p=*0.001). ^15^ Mahran *et al.*, 2000 reported significant differences regarding length of stay. It was shorter in the TUCL group (1.6 ± 0.8 days) than the CL group (3.9 ± 1.2 days) ( *p*<0.05). ^16^

Group C (PCCL *versus* CL)

Javanmard *et al.*, 2018 reported that the PCCL group (2.4 ± 0.7 days) had a shorter length of stay than the CL group (3.5 ± 1 days) ( *p=*0.001). ^15^ Mahran *et al.*, 2000 reported significant differences in terms of length of stay. It was shorter in the PCCL group (1.6 ± 0.8 days) than the CL group (3.9 ± 1.2 days) ( *p*<0.05). ^16^

### Results of syntheses

*Stone-free rate*

**Figure 3.**
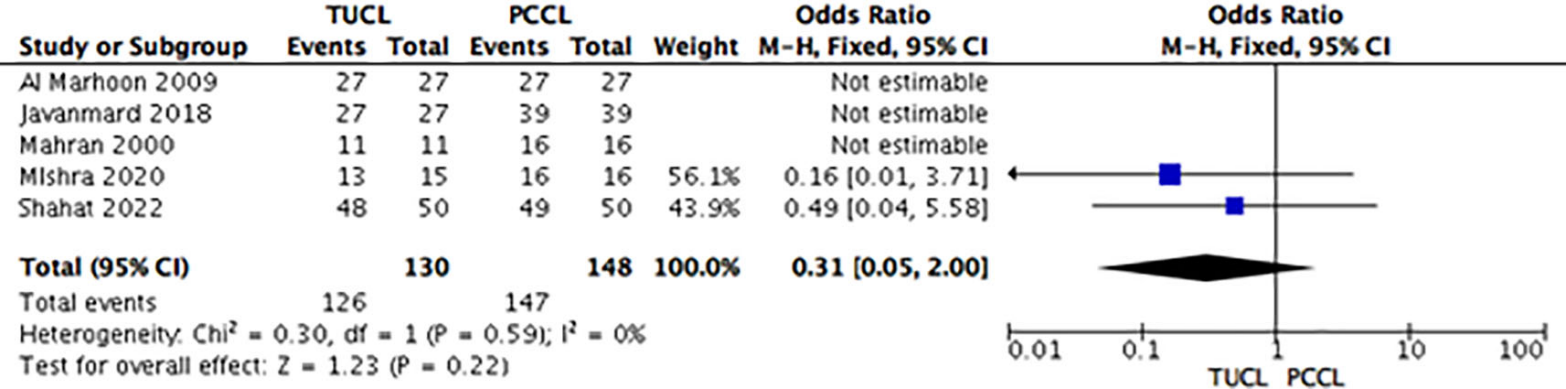
Forest plot of TUCL *vs.* PCCL stone-free rates. TUCL, transurethral cystolithotripsy; PCCL, percutaneous cystolithotomy.

All five studies reported the SFRs for all techniques. A meta-analysis for group A (TUCL *versus* PCCL) revealed no significant intergroup differences in SFR (OR 0.31; 95% CI: 0.05-2.00; p = 0.22; Figure 3). Meta-analysis was not deemed appropriate for other groups (TUCL *versus* PCCL and TUCL *versus* CL) because the heterogeneity test was not applicable ( Figure 3).

*Complications*

**Figure 4.**
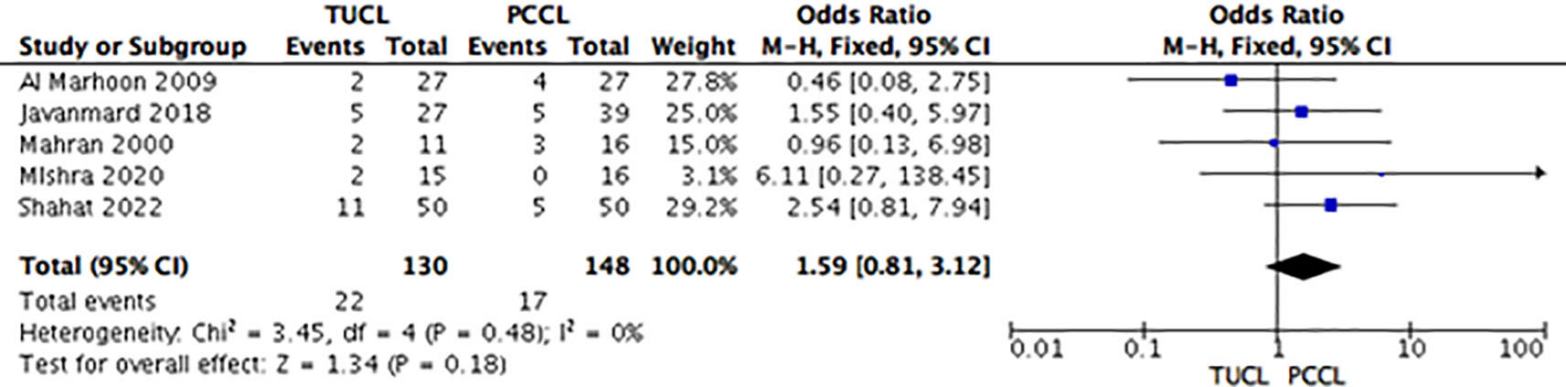
Forest plot of group A (TUCL *vs.* PCCL) complications. TUCL, transurethral cystolithotripsy; PCCL, percutaneous cystolithotomy.

**Figure 5.**
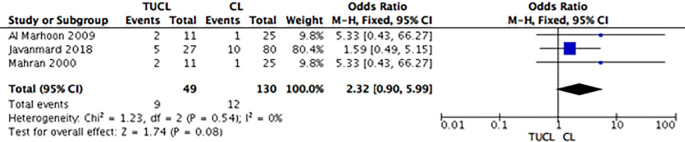
Forest plot of group B (TUCL *vs.* CL) complications. TUCL, transurethral cystolithotripsy; CL, cystolithotomy.

**Figure 6.**
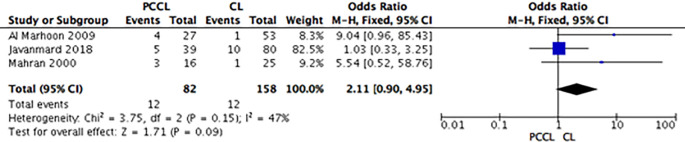
Forest plot of group C (PCCL *vs.* CL) complications. PCCL, percutaneous cystolithotomy; CL, cystolithotomy.

Complication rates were not significantly different among all studies in group A (TUCL *versus* PCCL) (OR: 1.59; 95% CI: 0.81-3.12; *p =* 0.18) ( Figure 4). Group B (TUCL *versus* CL) showed that the complication rate was lower among the CL group, but the intergroup differences were not significant (OR: 2.32; 95% CI: 0.90-5.99; *p =* 0.08) ( Figure 5). For group C (PCCL *versus* CL), the complication rate seemed lower among the CL patients, but the difference was not significant (OR: 2.11; 95% CI: 0.90-4.95; *p =* 0.09) ( Figure 6).

*Procedure duration*

**Figure 7.**
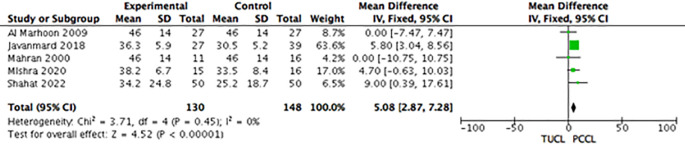
Forest plot of group A (TUCL *vs.* PCCL) procedure durations. TUCL, transurethral cystolithotripsy; PCCL, percutaneous cystolithotomy.

**Figure 8.**
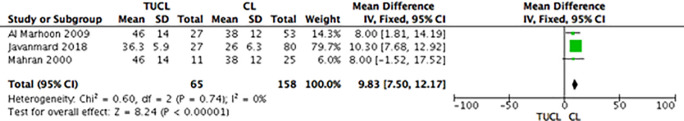
Forest plot of group B (TUCL *vs.* CL) procedure durations. TUCL, transurethral cystolithotripsy; CL, cystolithotomy.

**Figure 9.**
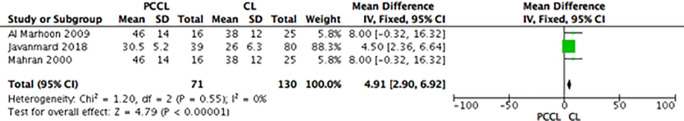
Forest plot of group C (PCCL *vs.* CL) procedure durations. PCCL, percutaneous cystolithotomy; CL, cystolithotomy.

For group A (TUCL *vs.* PCCL), the procedure duration was reported in all studies, and a meta-analysis showed a significant difference in favour of PCCL (Mean difference: 5.08; 95% CI: 2.87-7.28; *p* < 0.00001) ( Figure 7). Group B showed that the procedure duration of the CL group was shorter than that of the TUCL group (Mean Difference 9.83; 95% CI: 7.50-12.17; *p* < 0.00001) ( Figure 8). Group C reported that the CL group had a significantly shorter procedure duration than the PCCL group (Mean difference 4.91; 95% CI: 2.90-6.92; *p* < 0.0001) ( Figure 9).

*Length of hospital stays*

**Figure 10.**
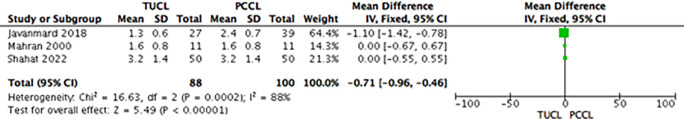
Forest plot of TUCL *vs.* PCCL lengths of hospital stay. TUCL, transurethral cystolithotripsy; PCCL, percutaneous cystolithotomy.

**Figure 11.**
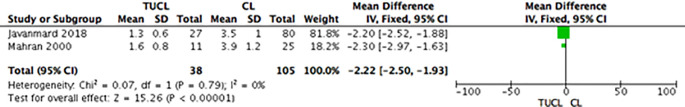
Forest plot of TUCL *vs.* CL lengths of hospital stay. TUCL, transurethral cystolithotripsy; CL, cystolithotomy.

**Figure 12.**
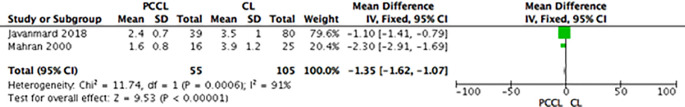
Forest plot of PCCL *vs.* CL lengths of hospital stay. PCCL, percutaneous cystolithotomy; CL, cystolithotomy.

Figure 10 shows that TUCL resulted in a significantly shorter length of hospital stay than PCCL (Mean difference -0.71; 95% CI: -0.96-(-0.46); *p* < 0.00001). For group B, the length of hospital stay was shorter in the TUCL group than the CL group (Mean difference -2.22; 95% CI: -2.50-(-1.93); *p* < 0.00001) ( Figure 11). Group C showed that the PCCL group had a significantly shorter hospital stay than the CL group (Mean difference: -1.35; 95% CI: -1.62-(-1.07); *p* < 0.00001) ( Figure 12).

### Reporting biases

As a rule of thumb, tests for funnel plot asymmetry should be used only when there are at least 10 studies included in the meta-analysis, because when there are fewer studies the power of the tests is too low to distinguish chance from real asymmetry. Thus, in this meta-analysis we did not proceed with reporting bias assessment.

### Certainty of evidence

This systematic review and meta-analysis have several limitations. In general, the quality of the studies according to the GRADE score in this meta-analysis varied from low to moderate. There were some studies with serious limitations and no reporting bias assessment was reported ( Table 2).

**Table 2.** GRADE evidence profile. NR, not reported; TUCL, transurethral cystolithotripsy; PCCL, percutaneous cystolithotomy; CL, cystolithotomy; NRS, non-randomised study; RCT, randomised controlled trials.

Quality assessment						
Outcome (no of studies and design; no of patients)	Study limitations	Inconsistency	Indirectness	Imprecision	Publication bias	Quality
Stone free rate						
• TUCL *versus* PCCL (4 NRS) (1 RCT) (437)	No serious limitation	No serious inconsistency	No serious indirectness	No serious imprecision	NR	Moderate
Complication						
• TUCL *versus* PCCL (4 NRS) (1 RCT) (437)	No serious limitation	No serious inconsistency	No serious indirectness	No serious imprecision	NR	Moderate
• TUCL *versus* CL (3NRS) (318)	Serious limitations	No serious inconsistency	No serious indirectness	No serious imprecision	NR	Low
• PCCL *versus* CL (3NRS) (318)	Serious limitations	No serious l inconsistency	No serious indirectness	No serious imprecision	NR	Low
Procedure duration						
• TUCL *versus* PCCL (4 NRS) (1 RCT) (437)	No serious limitation	No serious inconsistency	No serious indirectness	No serious imprecision	NR	Moderate
• TUCL *versus* CL (3 NRS) (318)	Serious limitations	No serious inconsistency	No serious indirectness	No serious imprecision	NR	Low
• PCCL *versus* CL (3 NRS) (318)	Serious limitations	No serious inconsistency	No serious indirectness	No serious imprecision	NR	Low
Length of stay						
• TUCL versus PCCL (2 NRS) (1 RCT) (311)	No serious limitation	No serious inconsistency	No serious indirectness	No serious imprecision	NR	Moderate
• TUCL *versus* CL (2 NRS) (211)	Serious limitations	No serious inconsistency	No serious indirectness	No serious imprecision	NR	Low
• PCCL *versus* CL (2 NRS) (211)	Serious limitations	No serious inconsistency	No serious indirectness	No serious imprecision	NR	Low

## Discussion

Urolithiasis in children is rare in the developed world, representing 1–5% of all cases of urinary tract stones. On the other hand, in developing countries, paediatric urolithiasis accounts for 30% of all cases of urinary tract stones, and the so-called endemic bladder stones are still common in childhood. In determining the treatment of choice for bladder stones, many factors must be considered, such as stone composition and size, general patient health, previous treatment history, and anatomic abnormalities. Surgical equipment and techniques also play an important role in therapeutic success. ^19^^,^^20^

Endoscopic techniques (TUCL and PCCL) are commonly used because of their high efficacy and low morbidity. ^21^ By contrast, the open surgical method (CL) has been used for a long time with high SFRs and low morbidity rates, and several urologists are familiar with this technique. However, whether one is superior in terms of operative characteristics or the incidence of complications, length of stay, and procedure duration, especially in children, remains unclear and controversial. Few studies have reported these data. Therefore, we conducted the present systematic review and meta-analysis to compare the efficacy and efficiency of these techniques in the removal of bladder stones in children. ^22^

Complete stone removal is the most important outcome in evaluations of the efficacy of stone treatment. The authors chose SFR as the main outcome of this study to help identify the best way to treat bladder stones in children. Our results indicated no difference in SFR among techniques, with SFR of 85–100%.

In terms of endoscopic treatment, there was no significant difference in SFR among procedures. Nevertheless, secondary outcomes were informative. PCCL was superior to TUCL in terms of procedure duration. Removing fragmented stones from the bladder is time-consuming in TUCL because of the narrow calibre of the urethra in children, and procedure time is the determining factor for relative complications and postoperative recovery in the TUCL procedure. ^23^^,^^24^ The risk of urethral stricture also increases due to urethral trauma during TUCL. ^14^^,^^25^ Ener *et al.*, assumed that the nephroscope used in PCCL has a larger lumen than the cystoscope, making it better for removing calculus fragments through its lumen, which can also shorten the procedure time. ^24^ In another case, TUCL had a shorter length of hospital stay than PCCL. We assumed that PCCL tended to have a longer hospital stay because of the wound that had been created to secure access to the bladder to remove the stones.

In comparing the endoscopic (TUCL and PCCL) *versus* CL approach, the CL technique had a shorter procedure duration; however, the endoscopic (TUCL and PCCL) techniques were superior in terms of length of hospital stay. The CL has an incision almost double that of the PCCL and TUCL, which expedited the bladder stone removal. Moreover, the operation success rate was 100%, but the involved wound led to an extended length of hospital stay *versus* the endoscopic techniques.

In addition to SFR, complication rates are important for treatment decisions. Major postoperative complication rates were low, and the meta-analysis revealed no significant differences among the techniques. However, CL was superior, with a lower complication rate than the endoscopic approach (TUCL and PCCL). Al-Marhoon *et al.*, showed that postoperative complications are increased with the percutaneous approach, reporting three cases of urinary leakage (5.6%) with the percutaneous approach *versus* one (1.9%) with the open approach. ^14^ Salah *et al.*, reported that complications of PCCL included paralytic ileus (9.7% patients) and abdominal distention (0.6%) due to the leakage of irrigation fluid into the abdominal cavity. ^25^ They reported that endourological management resulted in a shorter hospital admission but that CL was safer. ^14^^,^^25^ TUCL featured a longer procedure time than other approaches for larger or multiple stones, which might increase the risk of urethral stricture subsequent to urethral trauma in younger children due to the smaller urethral calibre. ^15^

This systematic review and meta-analysis included broad scope population (male and female children), which is also essential and could be implementable and applicable in clinical practice for treating bladder stones in children. Our meta-analysis indicates that further research comparing treatments for bladder stones, especially in children, is required to compare endoscopic treatments (TUCL and PCCL) and CL with more RCT-based data. Stone size and age may be added to the patient characteristics, which can be another informative outcome.

## Conclusions

This systematic review and meta-analysis showed that the three techniques (TUCL, PCCL, and CL) examined here had comparable SFRs. PCCL was superior to TUCL in terms of procedure duration; however, TUCL featured a shorter hospital stay than PCCL. Moreover, CL had a shorter procedure duration than either TUCL or PCCL; however, TUCL and PCCL featured shorter lengths of hospital stay than CL.

## Data Availability

All data underlying the results are available as part of the article and no additional source data are required. Figshare: PRISMA checklist for ‘Endourological *versus* open cystolithotomy for bladder stone management among children: A systematic review and meta-analysis’. https://doi.org/10.6084/m9.figshare.21915915. ^26^ Figshare: SAGER guidelines checklist for ‘Endourological versus open cystolithotomy for bladder stone management among children: A systematic review and meta-analysis’. https://doi.org/10.6084/m9.figshare.21922461. ^27^ Data are available under the terms of the Creative Commons Attribution 4.0 International license (CC-BY 4.0).
