# Supplementary material for: Endourological versus open cystolithotomy for bladder stone management among children: A systematic review and meta-analysis
Source: F1000Res. 2023 Feb 2;12:124. [Version 1] doi: 10.12688/f1000research.129270.1 (PMC11406119; doi:10.12688/f1000research.129270.1)
Supplement: Peer review history

# Reviewer report: Reviewer response for version 1

**DOI:** 10.5256/f1000research.141941.r184194

**Published:** 2024-09-16

**Authors:** Muhammad Asykar Palinrungi

**Affiliations:** 1 Universitas Hasanuddin, Makassar, South Sulawesi, Indonesia

Congratulation, you have successfully conducted a systematic review of this issue in detail and used a thorough analysis which has provided valuable insight to the scientific fields of pediatric urology. 

 I have one question: after you have explained the advantages and disadvantages of each action in this systematic review, what can you suggest or recommend that you might add to the conclusion?

Are the rationale for, and objectives of, the Systematic Review clearly stated?

Yes

Is the statistical analysis and its interpretation appropriate?

Yes

If this is a Living Systematic Review, is the ‘living’ method appropriate and is the search schedule clearly defined and justified? (‘Living Systematic Review’ or a variation of this term should be included in the title.)

Yes

Are sufficient details of the methods and analysis provided to allow replication by others?

Yes

Are the conclusions drawn adequately supported by the results presented in the review?

Yes

Reviewer Expertise:

paediatric urology, Functional Urology

I confirm that I have read this submission and believe that I have an appropriate level of expertise to confirm that it is of an acceptable scientific standard.

# Reviewer report: Reviewer response for version 1

**DOI:** 10.5256/f1000research.141941.r220407

**Published:** 2024-03-25

**Authors:** Rabea Ahmed Gadelkareem

**Affiliations:** 1 Urology Department, Assiut University, Asyut, Assiut Governorate, Egypt

The rationale and aim of this review are strong and clear. This systematic review was conducted using the PRISMA guidelines. It compared the techniques (TUCL, PCCL, and CL) for the treatment of bladder stones in children. The conclusion was based on sound methodology. It revealed that studies examined here showed comparable SFRs. PCCL was superior to TUCL in terms of procedure duration; however, TUCL featured a shorter hospital stay than PCCL. Moreover, CL had a shorter procedure duration than either TUCL or PCCL; however, TUCL and PCCL featured shorter lengths of  hospital stay than CL.

 However, some concerns should be addressed through the following comments:

 1) Abstract-Methods: Needs a more precise description of the methods

 2) Frame of search: from 1 to 30 August 2022!! It seems not accurately described in the text and abstract. You included studies between 2009 and 2022. So, this issue is a major concern because many points in the methods are based on it.

 3) Results synthesis; stone free rate, In the sentence (Meta-analysis was not deemed appropriate for other groups (TUCL *versus* PCCL and TUCL *versus* CL), there is a mistake in the groups: The group (TUCL *versus* PCCL) seems to be mistaken with the group (TUCL *versus* CL). Clarify.

 4) In the Discussion section: The grades and varieties of complications and their effects on the outcomes need more discussion. For example, were the intraperitoneal and intestinal injuries potential to all procedures? Also, were the urethral complications related to TUCL and affected its outcomes? Although these items were mentioned in the details of the Results, still their discussion can be improved.

Are the rationale for, and objectives of, the Systematic Review clearly stated?

Yes

Is the statistical analysis and its interpretation appropriate?

Yes

Are sufficient details of the methods and analysis provided to allow replication by others?

Partly

Are the conclusions drawn adequately supported by the results presented in the review?

Yes

Reviewer Expertise:

Endourology, urolithiasis, kidney transplantation

I confirm that I have read this submission and believe that I have an appropriate level of expertise to confirm that it is of an acceptable scientific standard, however I have significant reservations, as outlined above.

# Reviewer report: Reviewer response for version 1

**DOI:** 10.5256/f1000research.141941.r162181

**Published:** 2023-02-14

**Authors:** Andika Afriansyah

**Affiliations:** 1 RS Pusat Persahabatan, Jakarta, Indonesia

Congratulations on producing a well-executed systematic review. Your attention to detail and thorough analysis have undoubtedly contributed valuable insights to the existing body of knowledge in your field. The dedication to the rigorous process of conducting a systematic review is admirable, and your efforts have resulted in a comprehensive and reliable synthesis of the available evidence. 

 However, there are some points that you might be revised:

 1. Figures 10, 11, and 12 did not show clearly the *diamond *which shows the overall effect size.

 2. I don't know how to read clearly the meaning of table 2. I suggest the authors explain in more detail what table 2 want to describe especially the component of the column (study limitations, imprecision, etc).

 3. In the discussion, I suggest author consider the results of the meta-analysis based on the lithotripsy energy that used in the study.

Are the rationale for, and objectives of, the Systematic Review clearly stated?

Yes

Is the statistical analysis and its interpretation appropriate?

Yes

Are sufficient details of the methods and analysis provided to allow replication by others?

Yes

Are the conclusions drawn adequately supported by the results presented in the review?

Yes

Reviewer Expertise:

Endourology, Urooncology,  Functional Urology, Neurourology

I confirm that I have read this submission and believe that I have an appropriate level of expertise to confirm that it is of an acceptable scientific standard.
